# Useful Hints

**Published:** 1899-06

**Authors:** 


### Useful Hints.

Oxide of zinc for the final polishing of gold plates gives a beautiful polish, and does not soil the hands. The true working qualities of cohesive gold are best obtained by heating it on a sheet of mica. Direct heating in the flame of a spirit lamp injures its cohesiveness. When filling an incisor or cuspid that has a thin labial wall with cement or gutta percha, line the wall with four thicknesses of No. 4 gold-foil, it gives the tooth when finished a very nice appearance. In waxing up, oil the surface of wax before using blow-pipe to give final finish to case.—*Pharmaceutical Journal.*

